# Cyclic Imines (CIs) in Mussels from North-Central Adriatic Sea: First Evidence of Gymnodimine A in Italy

**DOI:** 10.3390/toxins12060370

**Published:** 2020-06-04

**Authors:** Simone Bacchiocchi, Melania Siracusa, Debora Campacci, Martina Ciriaci, Alessandra Dubbini, Tamara Tavoloni, Arianna Stramenga, Stefania Gorbi, Arianna Piersanti

**Affiliations:** 1 Istituto Zooprofilattico Sperimentale dell’Umbria e delle Marche “Togo Rosati”, Via Cupa di Posatora 3, 60131 Ancona, Italy; m.siracusa@izsum.it (M.S.); d.campacci@izsum.it (D.C.); m.ciriaci@izsum.it (M.C.); a.dubbini@izsum.it (A.D.); t.tavoloni@izsum.it (T.T.); a.stramenga@izsum.it (A.S.); a.piersanti@izsum.it (A.P.); 2 Dipartimento di Scienze della Vita e dell’Ambiente, Università Politecnica delle Marche, Via Brecce Bianche, 60131 Ancona, Italy; s.gorbi@univpm.it

**Keywords:** Adriatic Sea, gymnodimines (GYMs), spirolides (SPXs), bivalves, LC–MS/MS, *Mytilus galloprovincialis*, pinnatoxins (PnTXs), pteriatoxins (PtTXs)

## Abstract

Cyclic imines (CIs) are emerging marine lipophilic toxins (MLTs) occurring in microalgae and shellfish worldwide. The present research aimed to study CIs in mussels farmed in the Adriatic Sea (Italy) during the period 2014–2015. Twenty-eight different compounds belonging to spirolides (SPXs), gymnodimines (GYMs), pinnatoxins (PnTXs) and pteriatoxins (PtTXs) were analyzed by the official method for MLTs in 139 mussel samples collected along the Marche coast. Compounds including 13-desmethyl spirolide C (13-desMe SPX C) and 13,19-didesmethyl spirolide C (13,19-didesMe SPX C) were detected in 86% of the samples. The highest levels were generally reported in the first half of the year reaching 29.2 µg kg^−1^ in January/March with a decreasing trend until June. GYM A, for the first time reported in Italian mussels, was found in 84% of the samples, reaching the highest concentration in summer (12.1 µg kg^−1^). GYM A and SPXs, submitted to tissue distribution studies, showed the tendency to accumulate mostly in mussel digestive glands. Even if SPX levels in mussels were largely below the European Food Safety Authority (EFSA) reference of 400 μg SPXs kg^−1^, most of the samples contained CIs for the large part of the year. Since chronic toxicity data are still missing, monitoring is surely recommended.

## 1. Introduction

**Figure 1:**
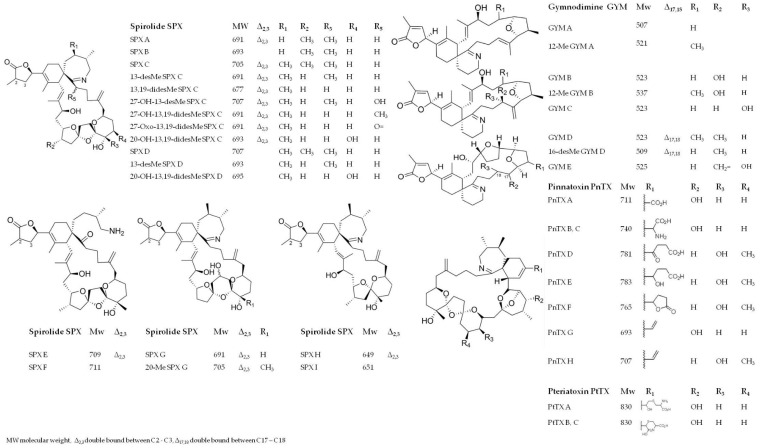
Chemical structures and the molecular information of the CI analogues identified in mussels and algae, reported in the literature so far.

Cyclic imines (CIs) are a group of marine lipophilic toxins (MLTs) originating from seawater phytoplankton, specifically from dinogflagellates. Spirolides (SPXs), gymnodimines (GYMs), pinnatoxins (PnTXs), pteriatoxins (PtTXs) are the major molecular groups belonging to CIs and the ones mostly detected in algae and mussels (Figure 1) [1].

Although rarely detected, other toxins are included in the CI group: prorocentrolides, spiro-prorocentrimine [1], symbioimines and portimine [2,3]. The cyclic imine moiety in the chemical structure of all these macrocyclic compounds is responsible for their fast acting toxicity in mice after intraperitoneal administration. The neurotoxic effects they induce are mostly a result of their specific interaction with the muscle and their neuronal nicotinic acetylcholine receptors [4].

CIs were discovered during the last 25 years because of their interference with the mouse bioassay for regulated MLTs in bivalve molluscs (okadaic acid, dinophysistoxins, pectenotoxins, yessotoxins, and azaspiracids) [5].

**Figure 2:**
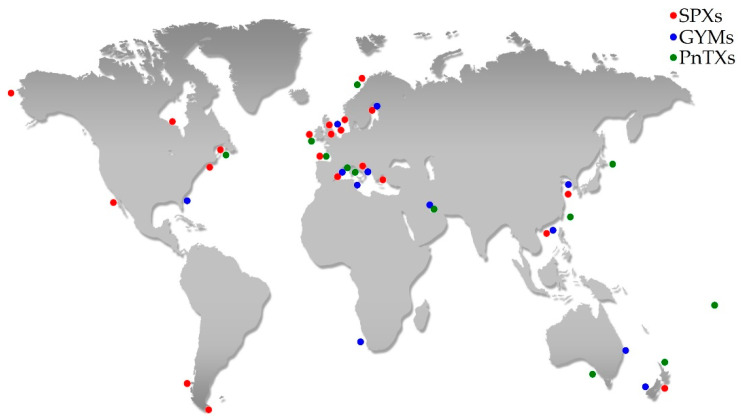
Worldwide SPX, GYM and PnTX distribution up to date.

SPXs were first detected in 1995, in shellfish from the Atlantic coast in Nova Scotia (Canada) [6] and subsequently, in phytoplankton and shellfish from all over the world (Figure 2) as promptly reported in Supplemental material Table S1 [7,8,9,10,11,12,13,14,15,16,17,18,19,20,21,22,23,24,25,26]. To date, 18 different SPX analogues have been isolated, representing the largest group of CIs (Figure 1) [27,28]. The dinoflagellates *Alexandrium ostenfeldii* and *A. peruvianum*, globally distributed species, are the only SPX-producer organisms known today [18,29,30]. At the end of November 2003, an *A. ostenfeldii* bloom was reported along the Emilia-Romagna coast in the North Adriatic Sea (Italy). On that occasion, mussels harvested during the algal bloom accumulated high levels of SPXs. The toxin profile of both the *A. ostenfeldii* and the mussels was characterized by 13-desmethyl spirolide C (13-desMe SPX C) and 13,19-didesmethyl spirolide C (13,19-didesMe SPX C) as the major components, with several minor spirolides like 27-hydroxy-13,19-didesmethyl spirolide C, 27-hydroxy-13-desmethyl spirolide C and 27-oxo-13,19-didesmethyl spirolide C [26,31].

In early 1990s, a first GYM analogue (GYM A) was isolated in oysters (*Tiostrea chilensis*) collected in South Island—New Zealand [32]. During that episode the dinoflagellate *Karenia selliformis* (formerly *Gymnodinium selliforme*) was identified as a GYM-producer organism [32]. Shortly afterwards, two different GYM analogues (GYM B and GYM C) were discovered in the cell cultures of New Zealand *K. selliformis* [33,34]. Today, eight GYM analogues have been identified (Figure 1) [28]. Besides the New Zealand episodes, GYMs have also been reported in many other countries (Table S1; Figure 2) [21,24,35,36,37,38,39,40]. Recently, it was demonstrated that the *A. ostenfeldii* and *A. peruvianum* possessed the ability to produce not only SPXs, but also GYMs [24,41,42].

PnTXs were first isolated from an extract of the shellfish species *Pinna attenuata* (Reeve, 1858) collected from Guangdong, China [43]. Overall, eight different analogues (from A until H; Figure 1) [1] were identified in shellfish and algae worldwide (Figure 2; Table S1) [43,44,45,46,47,48,49,50,51].

*V. rugosum* was shown to be able to produce PnTX E, F, G [50] and H [52]. PtTXs are scarcely studied members of the PnTX subgroup. PtTXs A, B and C (Figure 1) were isolated as extremely toxic components of *Pteria penguin* viscera in 2001 [53]. It was hypothesized that PnTX F and G may be precursors of all the other PnTXs and PtTXs throughout shellfish metabolic transformation (e.g., hydrolysis) [50].

Although CIs are known to occur in microalgae and/or shellfish worldwide, no human intoxication has been related to their presence in seafood so far [5,45,50,54]. No regulatory limits for CIs in shellfish have been set in Europe or the world to date, but the European Food Safety Authority (EFSA) requested more data to properly assess the risk CIs pose to shellfish consumers. The toxicology working group of the EU Community Reference Laboratory for Marine Biotoxins (CRLMB) proposed a tentative maximum limit alert of 400 μg of total SPXs kg^−1^ shellfish meat [55].

Given the above, the official LC–MS/MS method for regulated MLTs in bivalve molluscs [56] was extended also to the analysis of a wide range of CIs (SPXs, GYMs, PnTXs and PtTXs) to investigate their presence in *Mytilus galloprovincialis* harvested in the Adriatic Sea, along the Marche coast, during the period 2014–2015.

Overall, 139 samples were analyzed for the presence of 28 CI analogues in order to investigate seasonal trends and geographical distribution. These extensive monitoring results could enrich the European database on emerging marine toxins to represent the state of art on CI contamination in the North-Central Adriatic Sea, but on the other hand no other data were published about CIs in mussels since 2015. Considering the lack of information related to CI compartmentalization among shellfish tissues, a subset of samples was submitted to GYM A and SPX analysis to assess the amount of toxins accumulating in the digestive gland and the remaining flesh.

To the best of our knowledge, the present study is the first which undergoes extensive CI monitoring in mussels collected in the North-Central Adriatic Sea.

## 2. Results and Discussion

### 2.1. Method Performances Assessment

As already reported by Gerssen et al. [57] the EU-Harmonized Standard Operating Procedure (EU-SOP) for lipophilic marine biotoxins [56] enables us to analyze the CIs in the same analytical run used for the regulated MLTs. The developed method was able to reveal the CI presence in the mussels from the North-Central Adriatic Sea with good analytical performances. The limit of detection (LOD) and limit of quantification (LOQ) for the 13-desMe SPX C, GYM A and PnTX G was estimated to be 0.15 and 0.45 µg kg^−1^ respectively, suitable with respect to the guidance level (400 µg sum of SPX kg^−1^) proposed by the CRLMB.

The drift in the retention times was widely below 1%. The matrix matched the calibration curves and exhibited a good linearity for 13-desMe SPX C and GYM A with a correlation coefficient equal to or greater than 0.99. Response factors showed a drift < 10%, the residual analysis showed a random distribution over the entire concentration range.

Good recoveries, 92% and 94%, and acceptable repeatability-relative standard deviations (RSD_r_) of 8% and 5% were obtained for 13-desMe SPX C and GYM A respectively, on mussels spiked at 1 µg kg^−1^.

### 2.2. CIs in Mussels

**Figure 3:**
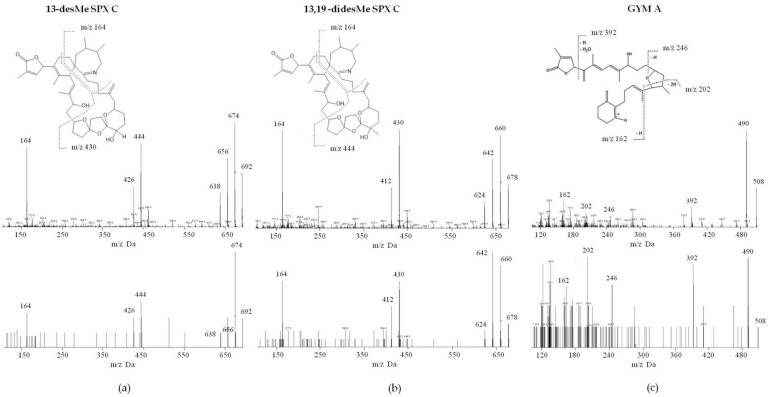
Collision-induced dissociation (CID) spectra of: (**a**) the 13-desMe SPX C; (**b**) the 13,19-didesMe SPX C; (**c**) the GYM A (top: reference standard, bottom: mussel sample).

The 13-desMe SPX C and the 13,19-didesMe SPX C were the only two SPX analogues found at detectable levels (> limit of detection, LOD = 0.15 µg kg^−1^) in 119 (86%) of the 139 mussel samples analyzed. The 13-desMe SPX C and the 13,19-didesMe SPX C were above the limit of quantification (LOQ = 0.45 µg kg^−1^) in 110 (79%) and in 108 (78%) samples, respectively (Table S2). The 13-desMe SPX C and 13,19-didesMe SPX C were unequivocally identified by comparing the collision-induced dissociation (CID) experiments on the contaminated mussels and the respective pure standards (Figure 3a,b).

**Figure 4:**
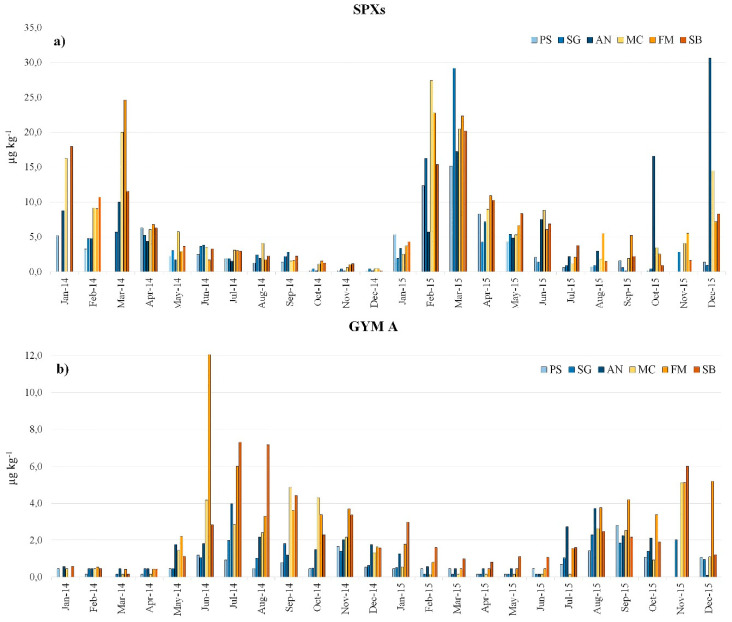
Total SPXs (sum of the 13-desMe SPX C and 13,19-didesMe SPX C in µg kg^−1^) (**a**) and the GYM A (µg kg^−1^) (**b**) seasonal trends in the six sampling stations PS (Pesaro), SG (Senigallia), AN (Ancona), MC (Macerata), FM (Fermo), and SB (San Benedetto del Tronto) along the Marche coast during the period 2014–2015.

The highest levels of SPXs (as a sum of the two analogues) were recorded in the first half of the year in both 2014 and 2015, in all the studied areas, with a maximum (29.2 µg kg^−1^) between January/March and then a decreasing trend until the end of the year (Figure 4a).

As an exception to this trend, unusually high SPX levels were recorded at the Ancona (AN) sampling site between October/December 2015. The observed general seasonal trend seemed to be different from those described for other MLTs in mussels from the North-Central Adriatic Sea such as okadaic acid and yessotoxins, which usually accumulate in shellfish during and or just after summer, respectively, reaching the highest levels in autumn [58].The latter trend fits perfectly with the well described phytoplankton dynamic in the Adriatic Sea, which includes two major annual biomass increases: the first in spring/early summer, corresponding to the general increased irradiance, water thermal stratification and nutrient availability in seawater, and the second in autumn, mainly caused by new nutrient input due to the increased inflow of freshwater from the Po River [59,60]. The SPX contamination of North-Central Adriatic Sea mussels, which reaches its maximum in winter, may not be explained by high plankton biomass blooms but probably it depends on the SPX primary producer’s ecophysiological characteristics: *A. ostenfeldii*, described in the North Adriatic Sea [61] is considered a cold-water species [7,62].

In some field studies, *A. ostenfeldii* was observed in coastal environments at temperatures ranging from 0.8 °C [63] to approximately 16–20 °C [64]; moreover, culture experiments with a Danish strain [65] and with a strain isolated from the Gulf of Maine [30] showed a better growth in cultures, at 16 and 14 °C, respectively.

**Figure 5:**
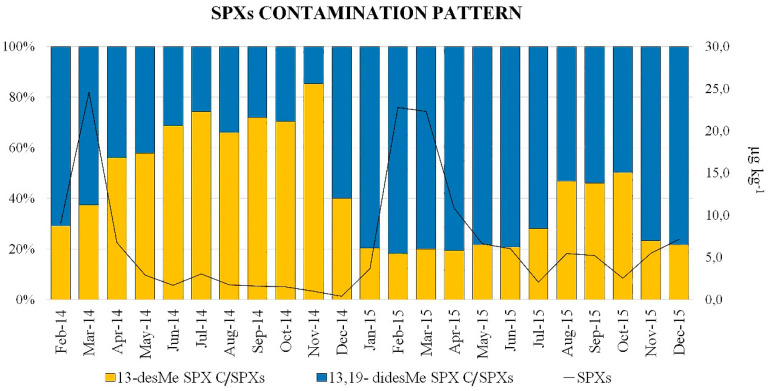
SPX contamination pattern in the FM sampling station: the 13-desMe SPX C percentage contribution in yellow and for 13,19-didesMe SPX C in blue. The line represents the concentration variation of the total SPXs in µg kg^−1^ during the period 2014–2015.

The SPX contamination pattern in mussels showed similar seasonal variations in all the coastal areas; as an example, in Figure 5 is reported the 13-desMe SPX C and 13,19-didesMe SPX C percentage contribution to the total SPX contamination in mussels from the Fermo (FM) area.

When the SPX concentration increased, the contamination pattern showed the 13,19-didesMe SPX C to be the analogue contributing the most (60–70%), while, as soon as the SPX concentration decreased, the profile in the mussels gradually changed toward a more consistent, or even predominant, contribution of the 13-desMe SPX C (Figure 5). The exception was found in the AN sampling site in October and December 2015, when highly contaminated mussels showed the 13-desMe SPX C to be the predominant SPX analogue (Table S2). The 13-desMe SPX C is generally reported as the analogue contributing the most to SPX contamination in European shellfish [55]. On the contrary, in the Emilia-Romagna coast, during 2003, 13,19-didesMe SPX C was the predominant analogue measured in shellfish [31]. The observed SPX contamination pattern variation in mussels may be a result of a different SPX analogue production by phytoplankton. SPX profiles in different *A. ostenfeldii* strains have been described in the literature [66,67,68] and even if the effect of environmental conditions on SPX composition is still controversial, some investigators demonstrated that SPX proportions can vary considerably depending on factors such as salinity, temperature and light intensity [66]. However, although 13-desMe SPX C was the most frequently observed analogue in the *A. ostenfeldii* toxin profile [20], the 13,19-didesMe SPX C, if present, resulted often predominant [65,66,69]. Another possible explanation for the SPX profile variation in mussels could be the different detoxification kinetics towards the individual SPX analogues, as a result of the different molecular structures and therefore, different mussel tissue affinity. GYM A, belonging to the GYM group, was the only analogue found at detectable levels (>LOD = 0.15 µg kg^−1^) in 117 (84%) of the 139 mussels analyzed. In 95 samples (68%) the contamination levels were above the LOQ (= 0.45 µg kg^−1^) (Table S2). The identity confirmation was accomplished by the CID experiments which unequivocally identified the GYM A in all the contaminated samples (Figure 3c). This was the first report of GYMs in Italian coastal waters and one of the few in Europe. These data also confirm the presence of GYMs in the Adriatic Sea as already reported in Croatian waters even if at concentrations below the LOQ [25]. The GYM A was detectable only in a few mussel samples in the first half of both years (2014 and 2015) started to increase, in all the areas, in early summer and reached maximum levels (12.1 µg kg^−1^) during summer/autumn to finally decrease in winter (Figure 4b). These temporal variations, unlike SPXs, seemed to agree with those described in mussels for other MLTs in the North-Central Adriatic Sea [58], probably as a result of the seasonal biomass growth dynamics. *K. selliformis*, the GYM-producing species, showed to be favored in its reproduction by high seawater temperature (>19 °C) [70], moreover, culture experiments involving a strain isolated from the Gulf of Gabès, Tunisia, showed that the highest growth rate was reached at 20 °C [71]. There are no records of *K. selliformis* in Italian waters, however the low levels of GYM A detected in mussels could have been produced by toxic microalgae cells present in the water at levels below the detection limit. Moreover, *A. ostenfeldii* and *A. peruvianum* have recently been reported to be GYM as well as SPX producers [24]; the culture experiment with an *A. ostenfeldii* strain isolated from the Baltic Sea showed that the highest GYM A concentration was reached at 20.9 °C [20]. Since *A. ostenfeldii* in the Adriatic Sea has been already reported by Pigozzi et al. [61], two different hypotheses on CI origin in mussels may be formulated. The first is that the 13-desMe SPX C, 13,19-didesMe SPX C and the GYM A originate from the same producer organism which varies the toxin profile as a result of the modified environmental conditions; the second holds that SPXs and GYM A are synthesized by different microalgae with different ecophysiological characteristics.

**Figure 6:**
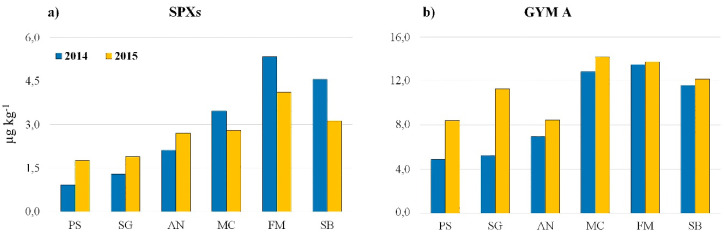
Total SPX (**a**) and GYM A (**b**) geographic distribution in the 6 sampling stations PS, SG, AN, MC, FM, SB during the period 2014–2015. The bars in the histogram represent the average concentration in the periods of maximum contamination: (**a**) Jun–Nov 2014, Aug–Nov 2015; (**b**) Jan–Apr 2014, Feb–Jun 2015.

The geographical trend of SPX and GYM A mussel contamination show that generally higher mean concentrations are reached in the southern Marche coasts (MC-Macerata, FM-Fermo, and SB-San Benedetto areas) during the maximum accumulation period (Figure 6), in contrast to what generally happens for other MLTs. Usually okadaic acid and yessotoxins affect earlier and more severely the northern part of the region with respect to the southern part, probably as a result of the stronger influence of the Po River delta, which, with its massive influx of nutrients, which represents the most important source of eutrophication in the Adriatic Sea [59]. CI producer proliferation along the Marche coast is probably more influenced by local eutrophication sources.

In general, CI contamination levels found in mussels from the Marche coast were in agreement with those reported in other geographical areas [13,14,15,16,17,18,19,20,21,22,23,24,25,26,72].

All the other CI groups like PnTXs and PtTXs were never detected (<LOD = 0.15 µg kg^−1^) in any of the mussel samples analyzed.

### 2.3. CI Compartmentalization in Mussels Tissues

**Figure 7:**
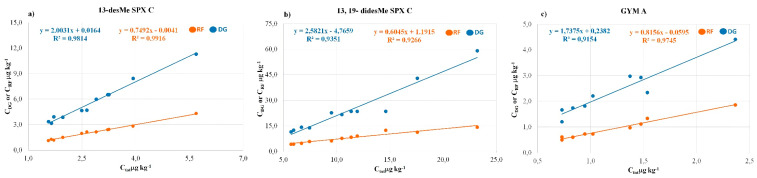
The 13-desMe SPX C (**a**), 13,19-didesMe SPX C (**b**) and the GYM A (**c**) concentrations in the digestive gland (DG) (blue) and the remaining flesh (RF) (orange) as a function of their own concentration in the whole flesh, in the analyzed mussels.

Overall, 11 samples for 13-desMe SPX C and 13,19-didesMe SPX C and nine for GYM A were submitted to compartmentalization studies, separately analyzing the CI concentration in the digestive gland (DG) and the remaining flesh (RF). In Figure 7a–c the concentration of the three CIs in the DG (blue) and the RF (orange) of the analyzed samples is represented as a function of their own concentration in the whole mussel flesh. Linear best-fit curves interpolating the data were plotted to obtain an acceptable correlation coefficient, meaning that both tissues’ toxin concentration increases proportionally with the increase in the overall CI concentration in the mussels. Although, generally for the same level measured in the whole tissue, the concentration in the DG is from two- (13-desMe SPX C and GYM A) to four-fold (13,19-didesMe SPX C) higher than in the RF, as shown by the best-fit curve slopes. The 13-desMe SPX C, 13,19-didesMe SPX C and GYM A behavior resulted in the uneven distribution of the CIs between the DG and the RF. Assuming a mussel composition of 20% by weight for the DG and 80% for the RF, roughly 40% of the toxins ingested by the mussel was found in the DG (Table S3). The preferential accumulation in the DG of the contaminated shellfish was also already reported for other marine biotoxins [73,74,75,76]. Moreover, our data agree with the results obtained by Medhioub et al. during the distribution studies conducted on *C. gigas* exposed to *A. ostenfeldii* [69].

## 3. Conclusions

The data collected in this study represent a very important insight into the state of the art on cyclic imine contamination of Adriatic Sea mussels. Despite the low levels, CIs were detected in more than 80% of the analyzed mussels for a large part of the year and it has been shown that environmental and climate conditions can strongly influence their accumulation in marine organisms. In this context, a continuous monitoring is surely recommended to be able to catch eventual changes, evaluating not only the acute toxicity towards humans, but also all the chronic, sub-chronic and possible synergistic effects. Acute toxicity towards mice has been already demonstrated, while the possible additive or reduced toxicological effects resulting from the simultaneous presence, in the same mussel sample, of different biotoxins should still be ascertained—hence the request for data on CIs in mussels from all over Europe issued by the EFSA in 2010. The biogenetic origin of the different Cyclic Imines found in the present study should be deeper investigated in order to identify the possible producers and their ecophysiological characteristics. The finding of gymnodimine A, for the first time being reported on the Italian coast of North-Central Adriatic Sea, enriches the knowledge on worldwide cyclic imine geographic distribution.

## 4. Materials and Methods

### 4.1. Chemicals and Standards

Acetonitrile (HPLC–MS CHROMASOLV) and ammonium hydroxide (≥25% in water, for trace analysis) were purchased from Fluka Analytical-Sigma-Aldrich (Steinheim, Germany). Methanol (CHROMASOLV, gradient grade for HPLC ≥ 99.9%), sodium hydroxide and hydrochloric acid were purchased from Sigma-Aldrich (Steinheim, Germany). Ultrapure water was produced by the MilliQ water purification system (Millipore Ltd., Bedford, MA, USA).

Certified solutions of 13-desMe SPX C (NRC CRM 13-desMe SPX C), PnTX G (NRC CRM PnTX-G) and GYM A (NRC CRM GYM) were purchased by the National Research Council Certified Reference Materials Program (Institute for Marine Biosciences, Halifax, NS, Canada) and used for quantification purposes. A non-certified standard solution of 13,19-didesMe SPX C (QCS 13,19-didesMeC) was purchased from CIFGA (Lugo, Spain) and used only for identification.

### 4.2. LC/MS-MS Analyses

The extraction was performed following the EU-SOP for lipophilic marine biotoxins in molluscs by LC–MS/MS [56] which also enabled the extraction of CIs together with the other regulated MLTs. The extract was finally analyzed by LC–MS/MS.

The chromatographic separation was achieved according to Gerssen et al. [57] following the analytical method described in Table S4.

Mass spectral experiments were performed using a hybrid triple-quadrupole/linear ion trap 3200 Q TRAP mass spectrometer (AB Sciex, Darmstadt, Germany) equipped with a Turbo V source and an electrospray ionization (ESI) probe. The mass spectrometer was coupled to a 1200-HPLC (Agilent—Palo Alto, CA, USA), which included an in-line degasser (G1379B), a quaternary pump (G1311A), a refrigerated autosampler (G1329A), and a column oven (G1316A).

The official LC–MS/MS procedure implemented for the analysis of marine lipophilic toxins since March 2012 was extended to include four groups of CIs: SPXs, GYMs, PnTXs and PtTXs.

The mass spectrometer was operated in multiple reaction monitoring (MRM) mode, selecting two transitions for each toxin to allow for quantification and identification (Table S4). The identification of 13-desMe SPX C, 13,19-didesMe SPX C, GYM A and PnTX G in the analyzed mussels was based on: retention times and ion ratios. In the case of all the other analytes, for which no reference materials were available, the identification was based on the characteristic transitions reported in literature. Only when the analytes were measured above the LOQ, in order to confirm the identification, were CID experiments on a linear ion trap (LIT) were conducted on samples and the respective pure standards. The ion source conditions for the MRM and the specific CID parameters described in Table S4 were used. In the quantification the matrix-matched calibration curves were used because of the non-negligible matrix effect. Assuming an equimolar response, analogues belonging to the SPX group were quantified with the analogue 13-desMe SPX C, GYMs with GYM A, PnTXs and PtTXs with PnTX G.

### 4.3. Analytical Method Performances Assessment

A double stage analytical method was implemented to make the monitoring of CIs easier and reliable. A qualitative screening procedure able to identify all the analytes belonging to the CI group present in the samples was set up using as reference standards 13-desMe SPX C, GYM A, PnTX G and 13,19-didesMe SPX C. The four reference materials were injected in the reported analytical conditions in order to set reference retention times. LODs were estimated only for the certified reference materials (13-desMe SPX C, GYM A, PnTX G), while 13,19-didesMe SPX C (not certified reference standard) was used only for confirmation in the CID experiments, being the most frequently quantified SPX analogue.

The performances of the quantitative screening procedure for the CIs were studied only for the toxins likely to be found in the samples analyzed using for quantification 13-desMe SPX C and GYM A as certified reference standards. Instrumental linearity was investigated by the matrix-matched calibration curves on five concentration levels (0.2, 0.3, 0.6, 1.0, 2.0 ng mL^−1^) for 13-desMe SPX C and GYM A and injected in triplicate the five calibration solutions. The calibration curves (*y = bx + a*) were obtained plotting the toxins chromatographic peak areas (*y*) against their concentrations (*x*). The best-fit curves were obtained using the least squares regression model. Linearity was evaluated from the correlation coefficients, response factors and residual analysis. The LODs were estimated according to the U.S. Environmental Protection Agency guidelines by multiplying the appropriate one-sided 99% t-statistic by the standard deviation [77]. This was obtained from six independently repeated analyses on a spiked matrix containing the analytes at a concentration three to five times higher than that of the estimated LOD. The LOQ was calculated multiplying the LOD value by 3.3. In our validation approach, the suitable standard deviations were those obtained from the experiments carried out at 1.0 µg kg^−1^. Accuracy in terms of recovery (R%) and precision (intra-day relative standard deviation RSDr%) were calculated performing replicated analyses (N = 6) on blank mussel samples spiked at 1.0 µg kg^−1^ with 13-desMe SPX C and 1.0 µg kg^−1^ GYM A. The spiked samples were quantified against the matrix-matched calibration curves. The drift in the retention times (RT) was considered acceptable if below 1%.

### 4.4. Experimental Plan

**Figure 8:**
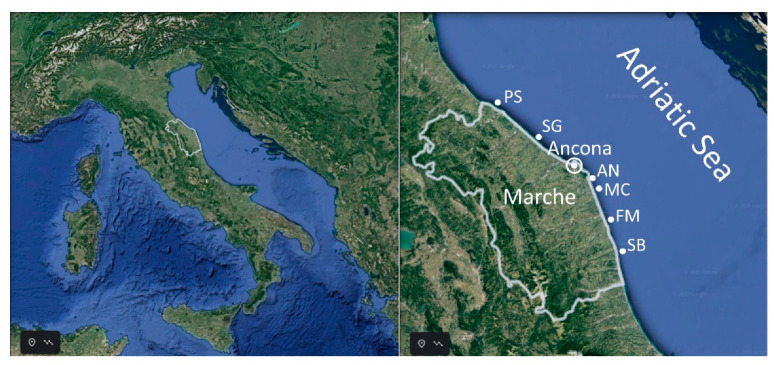
*Mytilus galloprovincialis* sampling sites along the Marche coast: PS (Pesaro), SG (Senigallia), AN (Ancona), MC (Macerata), FM (Fermo) and SB (San Benedetto del Tronto). Images (data SIO, NOAA, U.S. Navy, NGA, GEBCO; image Landsat/Copernicus) are from Google Earth.

Among all the authorized mussel harvesting sites included in the Marche biotoxins regional monitoring plan (North-Central Adriatic Sea; Italy), 6 breeding sites were selected for CI profile characterization. Samples of *M. galloprovincialis* were collected monthly in Pesaro (PS), Senigallia (SG), Ancona (AN), Macerata (MC), Fermo (FM) and San Benedetto del Tronto (SB) (Figure 8) covering the Marche coast from North to South. The sampling campaign started in January 2014 and ended in December 2015. Overall, 139 samples of mussels were collected and characterized for the presence of CIs.

The samples were immediately prepared once they arrived in the laboratory: the bivalves were opened, the sand and the solid residues removed under running water, and the mussels were taken out of the shells and drained on a net. For each sample, about 150 g of the whole tissue was pooled and finely homogenized and 2.0 ± 0.5 g was submitted for CI analysis. The rest of the homogenized tissue was stored at −20 °C.

During the year 2015, the mussel samples showing the highest CI contamination were submitted to further dissection, taking apart the digestive gland from the rest of the tissue. Pools of 10 specimens per sample were dissected and the analytical homogenates of the digestive gland (DG) and the remaining flesh (RF) were prepared and submitted for CI analysis in order to assess the toxin distribution. In the period of CI maximum levels, during the compartmentalization studies, the number of sampling sites was enlarged to also include other harvesting areas neighboring the ones selected.

## Supplementary Materials

The following are available online at https://www.mdpi.com/2072-6651/12/6/370/s1, Table S1: CI worldwide distribution, Table S2: GYM A, 13-desMe SPX C, 13,19-didesMe SPX C and sum of the two SPX analogues (∑ 2 SPXs) in the 139 mussel samples analyzed by LC–MS/MS, Table S3: 13-desMe SPX C, 13,19-dides Me SPX C and GYM A distribution in DG and RF (µgkg-1). C(DG)/C(RF) is the ratio between the concentrations. QCI(DG)/QCI(tot) (%) is the ratio between the µg of CI in DG and in the RF, in the hypothesis of a mussel composition of 20% by weight for DG and 80% for RF, Table S4: LC–MS/MS method for CI analysis: chromatographic conditions, MS parameters and transitions in multiple reaction monitoring (MRM). CID by LIT experimental conditions.
