# Supplementary material for: Cyclic Imines (CIs) in Mussels from North-Central Adriatic Sea: First Evidence of Gymnodimine A in Italy
Source: Toxins (Basel). 2020 Jun 4;12(6):370. doi: 10.3390/toxins12060370 (PMC7354633; doi:10.3390/toxins12060370)
Supplement: Supplementary file 1 [file toxins-12-00370-s001.pdf]

Supplementary Materials: Cyclic Imines (CIs) in Mussels from North-Central Adriatic Sea: First Evidence of Gymnodimine A in Italy

Simone Bacchiocchi, Melania Siracusa, Debora Campacci, Martina Ciriaci, Alessandra Dubbini, Tamara Tavoloni, Arianna Stramenga, Stefania Gorbi and Arianna Piersanti

Table S1. CIs worldwide distribution.

Geographic Area	Reference
**SPXs**	
Nova Scotia (Canada)	[6]
North America	[7,8]
South America	[9,10]
Bering Sea	[11]
China	[12,13]
New Zealand	[14]
Denmark	[15]
Norway	[16]
France	[17]
Ireland	[18]
Scotland	[19]
Baltic Sea	[20]
Spain (Catalonian coasts)	[21]
Spain (Galician coasts)	[22]
Greece	[23]
Holland	[24]
Croatia	[25]
Italy	[26]
**GYMs**	
New Zealand (South Island)	[32]
Australia	[35]
China	[36]
North America	[37]
Tunisia	[38]

South Africa	[39]
Qatar	[40]
Holland	[24]
Spain	[21]
Croatia	[25]
**PnTXs**	
China	[43]
Japan	[44]
New Zealand	[45]
Australia	[45]
Canada	[46]
CooK Islands	[50]
Qatar	[40]
Norway	[15]
Ireland	[47]
France	[48]
Spain	[49]
Italy	[51]

Table S2. GYM A, 13-desMe SPX C, 13,19-didesMe SPX C and sum of the two SPX analogues (SPXs) in the 139 mussel samples analysed by LC-MS/MS.

		**2014**												**2015**											
		**Jan**	**Feb**	**Mar**	**Apr**	**May**	**Jun**	**Jul**	**Aug**	**Sep**	**Oct**	**Nov**	**Dec**	**Jan**	**Feb**	**Mar**	**Apr**	**May**	**Jun**	**Jul**	**Aug**	Sep	Oct	Nov	Dec
		µg kg^{-1}																							
GYM-A	PS	<0.45	<0.15	NA*	<0.15	<0.45	1.19	0.93	0.46	0.79	<0.45	1.66	0.54	<0.45	<0.45	<0.45	<0.15	<0.15	<0.45	0.69	1.42	2.79	1.07	NA*	1.06
13-desMe SPX C	PS	2.07	0.83	NA*	3.79	1.48	1.49	1.32	0.82	0.96	<0.15	<0.15	<0.15	1.15	2.20	2.81	1.56	0.85	0.78	<0.15	0.55	0.86	<0.15	NA*	<0.15
13,19-didesMe SPX C	PS	3.10	2.43	NA*	2.49	0.80	0.96	0.53	<0.45	<0.45	<0.15	<0.15	<0.15	4.17	10.1	12.4	6.71	3.40	1.26	<0.45	<0.15	0.73	<0.15	NA*	1.23
SPXs	PS	5.17	3.26	NA*	6.29	2.28	2.45	1.85	1.27	1.41	<0.15	<0.15	<0.15	5.32	12.3	15.2	8.27	4.25	2.04	0.60	0.70	1.58	<0.15	NA*	1.38
GYM-A	SG	NA*	<0.45	<0.15	<0.45	<0.45	1.05	1.99	1.03	1.81	0.49	1.41	0.63	0.51	<0.15	<0.15	<0.15	<0.15	<0.15	1.04	2.29	1.86	1.40	2.02	0.95
13-desMe SPX C	SG	NA*	1.29	2.09	2.77	1.74	2.46	1.32	1.74	1.64	<0.15	<0.15	<0.15	<0.45	3.36	4.89	1.25	1.20	<0.45	<0.45	<0.45	<0.15	<0.15	<0.45	<0.15
13,19-didesMe SPX C	SG	NA*	3.47	3.58	2.44	1.30	1.21	0.54	0.67	0.56	<0.15	<0.15	<0.15	1.44	12.9	24.3	2.97	4.16	0.93	<0.45	<0.45	<0.45	<0.15	2.38	0.76
SPXs	SG	NA*	4.76	5.67	5.21	3.04	3.67	1.86	2.41	2.20	0.40	0.40	0.40	1.89	16.3	29.2	4.22	5.36	1.38	0.90	0.90	0.60	0.40	2.83	0.91
GYM-A	AN	0.56	<0.45	<0.45	<0.45	1.77	1.81	3.97	2.18	1.19	1.48	2.03	1.75	1.27	0.57	<0.45	<0.45	<0.45	<0.15	2.72	3.72	2.24	2.12	NA*	0.10
13-desMe SPX C	AN	3.27	1.25	4.62	1.90	1.01	2.44	1.04	1.27	2.22	<0.15	<0.15	<0.15	0.82	1.33	2.55	1.03	1.46	2.24	0.78	1.42	<0.15	16.4	NA*	30.5
13,19-didesMe SPX C	AN	5.48	3.53	5.36	2.43	0.72	1.38	0.47	0.64	0.60	<0.15	<0.15	<0.15	2.53	4.36	14.7	6.12	3.35	5.26	1.37	1.55	<0.15	<0.15	NA*	<0.15

SPXs		8.75	4.78	9.98	4.33	1.73	3.82	1.52	1.91	2.82	<0.15	<0.15	<0.15	3.34	5.69	17.2	7.15	4.80	7.50	2.15	2.96	<0.15	16.6	NA*	30.6
GYM-A		<0.45	<0.45	<0.15	<0.15	1.44	4.19	2.85	2.40	4.88	4.31	2.17	1.31	0.54	0.14	<0.15	<0.15	<0.15	<0.15	<0.15	2.62	2.53	0.92	5.12	1.09
13-desMe SPX C		4.40	2.45	6.85	2.88	2.98	2.37	2.25	2.89	1.07	0.95	<0.15	<0.15	0.40	3.40	3.73	1.45	0.87	2.78	<0.45	1.05	0.43	1.75	0.69	1.53
13,19-didesMe SPX C	MC	11.8	6.71	13.1	3.17	2.75	1.13	0.87	1.16	0.47	<0.15	<0.45	0.10	2.09	24.0	16.7	7.52	4.42	6.03	0.73	0.74	1.47	1.67	3.36	12.9
SPXs		16.2	9.16	20.0	6.06	5.73	3.50	3.12	4.05	1.53	1.10	0.60	0.40	2.49	27.4	20.4	8.98	5.30	8.81	1.18	1.79	1.90	3.42	4.04	14.5
GYM-A		NA*	0.53	0.42	0.43	2.20	12.1	6.00	3.29	3.61	3.38	3.70	1.62	1.79	0.79	<0.45	<0.45	<0.45	<0.45	1.56	3.76	4.20	3.38	5.12	5.19
13-desMe SPX C		NA*	2.64	9.21	3.80	1.67	1.17	2.26	1.14	1.16	1.08	0.87	0.10	0.75	4.14	4.47	2.12	1.44	1.26	0.59	2.56	2.41	1.27	1.29	1.55
13,19-didesMe SPX C	FM	NA*	6.42	15.4	2.98	1.21	0.53	0.78	0.59	<0.45	<0.45	<0.15	<0.15	2.93	18.6	17.9	8.76	5.20	4.78	1.50	2.90	2.82	1.26	4.23	5.58
SPXs		NA*	9.05	24.6	6.79	2.89	1.70	3.04	1.73	1.61	1.53	1.02	0.40	3.68	22.8	22.3	10.9	6.64	6.04	2.09	5.46	5.23	2.53	5.51	7.13
GYM-A		0.57	<0.45	<0.15	0.43	1.13	2.83	7.30	7.18	4.42	2.28	3.37	1.58	2.98	1.60	0.98	0.81	1.11	1.06	1.60	2.46	2.16	1.89	6.00	1.21
13-desMe SPX C		5.86	2.62	6.09	3.09	2.04	1.79	1.95	1.64	1.46	0.81	0.59	<0.15	1.45	2.64	4.22	2.28	2.18	2.41	1.36	0.52	0.77	<0.45	0.56	1.35
13,19-didesMe SPX C	SB	12.1	8.08	5.41	3.19	1.60	1.51	0.99	0.63	0.76	<0.45	0.54	<0.15	2.86	12.7	16.0	7.97	6.13	4.43	2.41	0.94	1.42	<0.45	1.06	6.90
SPXs		18.0	10.7	11.5	6.27	3.64	3.30	2.94	2.27	2.22	1.26	1.13	<0.15	4.32	15.4	20.2	10.2	8.31	6.84	3.77	1.46	2.19	0.90	1.62	8.26

NA*= not analysed.

Table 3. 13-desMe SPX C, 13,19-dides Me SPX C and GYM A distribution in DG and RF (μgkg^{-1}). C(DG)/C(RF) is the ratio between the concentrations. Q_{CI}(DG)/Q_{CI}(tot) (%) is the ratio between the ⊚g of CI in DG and in the RF, in the hypothesis of a mussel composition of 20% by weight for DG and 80% for RF.

	C tot (μg/kg)	C(DG) (μg/kg)	C(RF) (μg/kg)	C(DG)/C(RF)	Q_{CI}(DG)/Q_{CI}(tot) %
			**13-desMe SPX C**		
**mean**	**2.82**	5.66	2.11	2.7	**40**
**median**	**2.64**	4.68	2.13	2.2	**40**
**min**	**1.56**	3.15	1.12	2.8	**36**
**max**	**5.69**	11.3	4.29	2.6	**46**
			**13,19-didesMe SPX C**		
**mean**	**11.31**	24.4	8.03	3.0	**42**
**median**	**10.48**	22.7	7.68	2.9	**41**
**min**	**5.70**	11.5	4.26	2.7	**32**
**max**	**23.20**	59.1	14.2	4.2	**51**

	**GYM A**				
**mean**	**1.22**	2.36	0.94	2.5	**39**
**median**	**1.02**	2.21	0.73	3.0	**40**
**min**	**0.73**	1.20	0.50	2.4	**30**
**max**	**2.37**	4.41	1.86	2.4	**46**

Table S4. LC-MS/MS method for CIs analysis: chromatographic conditions, MS parameters and transitions in multiple reaction monitoring (MRM). CID by LIT[c] experimental conditions.

LC PARAMETERS

		**Time (min)**	**A (%)**	**B (%)**
**Column Type**	X-Bridge™ C18 5◎◎m, 3.0 x 150 mm (Waters)			
**Injection Volume**	10 µL	0.0	90	10
**Flow**	0.4 mL/min	2.0	90	10
**Column temperature**	40°C	13.0	10	90
**Mobile phase A**	0.05% v/v NH_4OH in H_2O (~ pH 11)	18.0	10	90
**Mobile phase B**	0.05% v/v NH_4OH in CH_3CN : H_2O (90:10) (~pH 11)	21.0	90	10
		27.0	90	10

MS/MS PARAMETERS

**Source type**	ESI	**Source temperature**	600 °C		
**Collision gas**	Medium			**Curtain gas**	20 psi
**IonSpray voltage**	5000 V	**Ion source Gas 1**	60 psi	**Ion source Gas 2**	50 psi

MRM TRANSITIONS

Toxin	Prec. ion (m/z)	Prod. ion (m/z)	CE [a] (V)	Toxin	Prec. ion (m/z)	Prod. ion (m/z)	CE [a] (V)
**SPX A**	692 [b]	444	40	**SPX H**	650 [b]	402	40
		150	45			164	45
**SPX B**	694 [b]	444	40	**SPX I**	652 [b]	402	40
		150	45			164	45
**SPX C**	706 [b]	458	40	**PnTX A**	712 [b]	458	45
		164	45			164	55
**13-desMe SPX C**	692 [b]	444	40	**PnTX B/C**	741 [b]	458	45

		164	45			164	55
**27 OH -13-desMe SPX C**	708 [b]	460	40	**PnTX D**	782 [b]	488	45
		180	45			164	55
**13, 19-didesMe SPX C**	678[b]	430	40	**PnTX E**	784 [b]	488	45
		164	45			164	55
**27 OH-13, 19-desMe SPX C**	694 [b]	446	40	**PnTX F**	766 [b]	488	45
		180	45			164	55
**27 Oxo-13, 19-desMe SPX C**	692 [b]	444	40	**PnTX G**	694 [b]	458	45
		178	45			164	55
**SPX D**	708 [b]	458	40	**PtTX A/B/C**	831 [b]	458	45
		164	45			164	55
**13-desMe SPX D**	694 [b]	444	40	**GYM A**	508 [b]	490	40
		164	45			162	45
**SPX G**	692 [b]	378	40	**12 Me GYM A**	522 [b]	504	40
		164	45			162	45
**20-Me SPX G**	706 [b]	392	40	**GYM B/C**	524 [b]	506	40
		164	45			162	45

CID EXPERIMENTS (LIT [c])

Scan speed 1000 Da/s **Dynamic fill time**

**Toxin**	**Precursor ion (m/z)**	**Mass range (m/z)**	**CE [a] (V)**
**13-desMe SPX C**	692 [b]	100–695	55
**13, 19-didesMe SPX C**	678 [b]	100–680	55
**GYM A**	508 [b]	100–510	45

[a] CE = Collision Energy, [b] $[M + H]^+$, [c] LIT= Linear ion trap.
